# 14-Eth­oxy-4,6,9-trimethyl-8,12-dioxa-4,6-diaza­tetra­cyclo­[8.8.0.0^2,7^.0^13,18^]octa­deca-2(7),13,15,17-tetra­ene-3,5,11-trione

**DOI:** 10.1107/S1600536813000743

**Published:** 2013-01-23

**Authors:** G. Jagadeesan, D. Kannan, M. Bakthadoss, S. Aravindhan

**Affiliations:** a Department of Physics, Presidency College, Chennai 600 005, India; b Department of Organic Chemistry, University of Madras, Guindy Campus, Chennai 600 025, India

## Abstract

In the title compound, C_19_H_20_N_2_O_6_, the pyrone and pyran rings adopt envelope conformations with the same common C atom as the flap, the dihedral angle between the planes of the remaining ring atoms being 68.27 (4)°. The planar atoms of the pyran ring and the diaza­cyclic ring are almost coplanar, the dihedral angle between their mean planes being 3.29 (7)°. Moreover, the planar atoms of the pyrone ring and benzene ring of the coumarin unit are also close to coplanar, the dihedral angle between their mean planes being 8.03 (9)°. The meth­oxy group lies in the plane of the benzene ring, with a dihedral angle between their mean planes of 9.4 (2)°. In the crystal, the molecules are linked by C—H⋯O hydrogen bonds resulting in sheets of mol­ecules in the *ac* plane.

## Related literature

For the biological activity of pyran­ocoumarin compounds, see: Kawaii *et al.* (2001 ▶); Goel *et al.* (1997 ▶); Su *et al.* (2009 ▶). For a related structure, see: Pojarová *et al.* (2012 ▶).
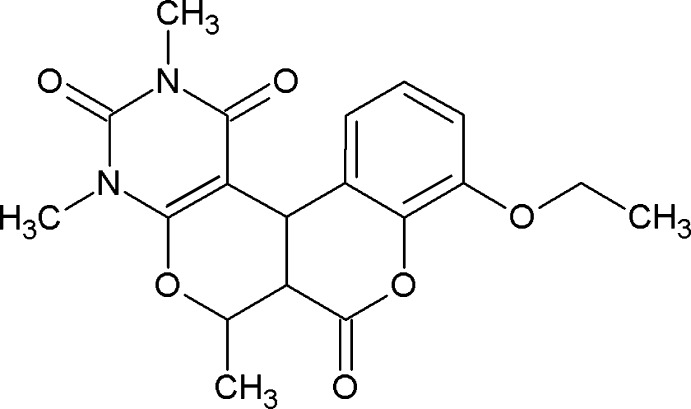


## Experimental

### 

#### Crystal data

C_19_H_20_N_2_O_6_
*M* *_r_* = 372.37
Monoclinic, 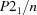

*a* = 9.3526 (3) Å
*b* = 17.9559 (5) Å
*c* = 10.9158 (3) Å
β = 101.346 (1)°
*V* = 1797.31 (9) Å^3^
*Z* = 4
Mo *K*α radiation
μ = 0.10 mm^−1^
*T* = 293 K
0.25 × 0.20 × 0.20 mm

#### Data collection

Bruker Kappa APEXII CCD diffractometer
Absorption correction: multi-scan (*SADABS*; Bruker 2004 ▶) *T*_min_ = 0.979, *T*_max_ = 0.983
21571 measured reflections
5615 independent reflections
3743 reflections with *I* > 2σ(*I*)
*R*_int_ = 0.024

#### Refinement

*R*[*F* ^2^ > 2σ(*F* ^2^)] = 0.049
*wR*(*F* ^2^) = 0.151
*S* = 1.03
5615 reflections
245 parameters
H-atom parameters constrained
Δρ_max_ = 0.31 e Å^−3^
Δρ_min_ = −0.20 e Å^−3^

### 

Data collection: *APEX2* (Bruker, 2004 ▶); cell refinement: *APEX2* and *SAINT* (Bruker, 2004 ▶); data reduction: *SAINT* and *XPREP* (Bruker, 2004 ▶); program(s) used to solve structure: *SHELXS97* (Sheldrick, 2008 ▶); program(s) used to refine structure: *SHELXL97* (Sheldrick, 2008 ▶); molecular graphics: *ORTEP-3 for Windows* (Farrugia, 2012 ▶); software used to prepare material for publication: *PLATON* (Spek, 2009 ▶).

## Supplementary Material

Crystal structure: contains datablock(s) I, global. DOI: 10.1107/S1600536813000743/pv2613sup1.cif

Structure factors: contains datablock(s) I. DOI: 10.1107/S1600536813000743/pv2613Isup2.hkl

Supplementary material file. DOI: 10.1107/S1600536813000743/pv2613Isup3.cml

Additional supplementary materials:  crystallographic information; 3D view; checkCIF report

## Figures and Tables

**Table 1:** Hydrogen-bond geometry (Å, °)

*D*—H⋯*A*	*D*—H	H⋯*A*	*D*⋯*A*	*D*—H⋯*A*
C2—H2⋯O6^i^	0.98	2.40	3.1480 (17)	132
C18—H18*C*⋯O3^ii^	0.96	2.45	3.252 (2)	140

Symmetry codes: (i) 
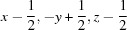
; (ii) 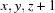.

**Fig. 1.**
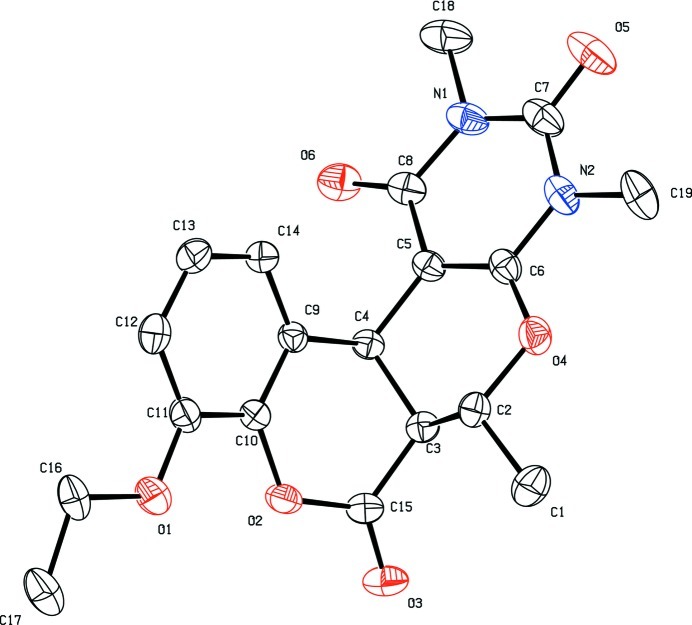
The molecular structure of the title compound, Displacement ellipsoids are drawn at the 30% probability level, H atoms have been omitted for clarity.

**Fig. 2.**
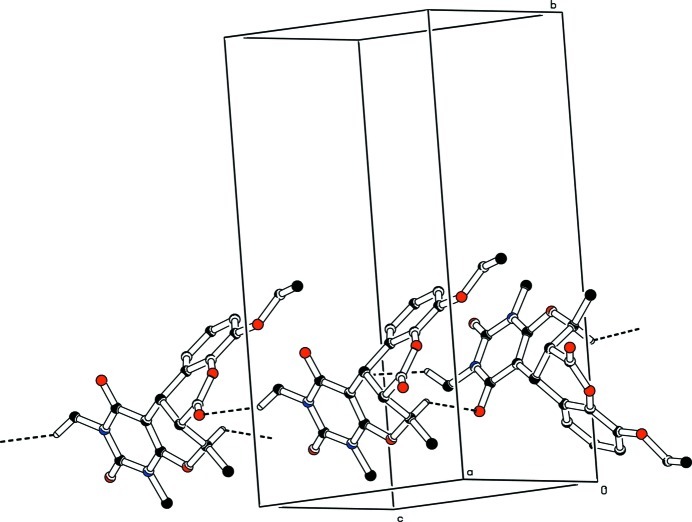
Crystal packing of the title compound, Hydrogen bonds are shown as dashed lines. For the sake of clarity, H atoms not involved in the interactions have been omitted.

## supplementary crystallographic information

### Comment

Coumarin derivatives show strong activity against cancer cell lines (Kawaii *et al.*, 2001) and exhibit antiulcer (Goel *et al.*, 1997) and cytotoxic activities (Su *et al.*, 2009). We report here in this paper the crystal structure of the title coumarin derivative.

In the title molecule (Fig. 1), the pyrone (O2/C3/C4/C9/C10/C15) and pyran (O4/C2–C6) rings adopt C3-envelope conformations with C3 displaced by 0.603 (2) and 0.668 (2) Å, respectively, from the least-square planes formed by the remaining ring atoms; the dihedral angle between the two mean-planes being 68.27 (4)°. The planar atoms of the pyran ring (O4/C2/C4–C6) and diazacyclic ring (N1/N2/C5–C8) are almost co-planar with dihedral angle between the mean-planes being 3.29 (7)°. Moreover, the planar atoms of the pyrone ring (O2/C4/C9/C10/C15) and benzene ring (C9–C14) of the coumarin moiety are also co-planar with dihedral angle between the mean-planes being 8.03 (9)°. The methoxy group (O1/C16/C17) lies in the plane of the benzene ring (C9–C14) with a dihedral angle between the mean-planes 9.4 (2)°. The crystal packing is stabilized by intermolecular C2—H2···O6 and C18—H18C···O3 hydrogen bonding interactions (Fig. 2 and Table 1).

### Experimental

A mixture of 2-ethoxy-6-formylphenyl (2E)-but-2-enoate (0.234 g, 1 mmol) and *N*,*N*-dimethylbarbituric acid (0.156 g, 1 mmol) was placed in a round bottom flask and melted at 14-Ethoxy-4,6,9-trimethyl-8,12-dioxa-4,6-diazatetracyclo[8.8.0.0^2,7^.0^13,18^]octadeca-2(7),13,15,17-tetraene-3,5,11-trione180 °C for 1 h. After completion of the reaction as indicated by TLC, the crude product was washed with 5 ml of ethylacetate and hexane mixture (1:49 ratio) which successfully provided the pure product in 90% yield as colorless solid.

### Refinement

All the H atoms were positioned geometrically, with C–H = 0.93–0.97 A and constrained to ride on their parent atom, with *U*_iso_(H) =1.5Ueq for methyl H atoms and 1.2Ueq(C) for other H atoms.

### Crystal data

**Table:**

C_19_H_20_N_2_O_6_	*F*(000) = 784
*M**_r_* = 372.37	*D*_x_ = 1.376 Mg m^−^^3^
Monoclinic, *P*2_1_/*n*	Mo *K*α radiation, λ = 0.71073 Å
Hall symbol: -P 2yn	Cell parameters from 8834 reflections
*a* = 9.3526 (3) Å	θ = 2.1–31.2°
*b* = 17.9559 (5) Å	µ = 0.10 mm^−^^1^
*c* = 10.9158 (3) Å	*T* = 293 K
β = 101.346 (1)°	Block, colourless
*V* = 1797.31 (9) Å^3^	0.25 × 0.20 × 0.20 mm
*Z* = 4	

### Data collection

**Table:**

Bruker Kappa APEXII CCD diffractometer	5615 independent reflections
Radiation source: fine-focus sealed tube	3743 reflections with *I* > 2σ(*I*)
Graphite monochromator	*R*_int_ = 0.024
ω and φ scan	θ_max_ = 31.2°, θ_min_ = 2.2°
Absorption correction: multi-scan (*SADABS*; Bruker 2004)	*h* = −13→13
*T*_min_ = 0.979, *T*_max_ = 0.983	*k* = −26→25
21571 measured reflections	*l* = −15→9

### Refinement

**Table:**

Refinement on *F*^2^	Secondary atom site location: difference Fourier map
Least-squares matrix: full	Hydrogen site location: inferred from neighbouring sites
*R*[*F*^2^ > 2σ(*F*^2^)] = 0.049	H-atom parameters constrained
*wR*(*F*^2^) = 0.151	*w* = 1/[σ^2^(*F*_o_^2^) + (0.0701*P*)^2^ + 0.3178*P*] where *P* = (*F*_o_^2^ + 2*F*_c_^2^)/3
*S* = 1.03	(Δ/σ)_max_ < 0.001
5615 reflections	Δρ_max_ = 0.31 e Å^−^^3^
245 parameters	Δρ_min_ = −0.20 e Å^−^^3^
0 restraints	Extinction correction: *SHELXL97* (Sheldrick, 2008), Fc^*^=kFc[1+0.001xFc^2^λ^3^/sin(2θ)]^-1/4^
Primary atom site location: structure-invariant direct methods	Extinction coefficient: 0.0038 (11)

### Special details

**Table:**

Geometry. All e.s.d.'s (except the e.s.d. in the dihedral angle between two l.s. planes) are estimated using the full covariance matrix. The cell e.s.d.'s are taken into account individually in the estimation of e.s.d.'s in distances, angles and torsion angles; correlations between e.s.d.'s in cell parameters are only used when they are defined by crystal symmetry. An approximate (isotropic) treatment of cell e.s.d.'s is used for estimating e.s.d.'s involving l.s. planes.
Refinement. Refinement of *F*^2^ against ALL reflections. The weighted *R*-factor *wR* and goodness of fit *S* are based on *F*^2^, conventional *R*-factors *R* are based on *F*, with *F* set to zero for negative *F*^2^. The threshold expression of *F*^2^ > σ(*F*^2^) is used only for calculating *R*-factors(gt) *etc*. and is not relevant to the choice of reflections for refinement. *R*-factors based on *F*^2^ are statistically about twice as large as those based on *F*, and *R*- factors based on ALL data will be even larger.

### Fractional atomic coordinates and isotropic or equivalent isotropic displacement parameters (Å^2^)

**Table:**

	*x*	*y*	*z*	*U*_iso_*/*U*_eq_	
C4	0.13936 (13)	0.22959 (7)	0.17644 (11)	0.0356 (3)	
H4	0.2334	0.2050	0.2061	0.043*	
O2	0.12546 (11)	0.18881 (6)	−0.07686 (9)	0.0488 (3)	
O4	−0.00265 (11)	0.37272 (5)	0.16207 (10)	0.0509 (3)	
C3	0.15760 (13)	0.28831 (7)	0.07958 (12)	0.0385 (3)	
H3	0.2385	0.3211	0.1163	0.046*	
C5	0.09372 (13)	0.26778 (7)	0.28399 (12)	0.0383 (3)	
N1	0.07800 (14)	0.27406 (8)	0.50011 (11)	0.0533 (3)	
O3	0.27612 (11)	0.27910 (7)	−0.09473 (10)	0.0570 (3)	
O6	0.18464 (12)	0.17436 (7)	0.42755 (10)	0.0582 (3)	
C9	0.03042 (13)	0.17227 (7)	0.11316 (12)	0.0359 (3)	
N2	−0.01928 (14)	0.37032 (8)	0.36503 (13)	0.0546 (3)	
C2	0.01853 (15)	0.33507 (7)	0.04908 (13)	0.0423 (3)	
H2	−0.0645	0.3019	0.0203	0.051*	
C6	0.02593 (14)	0.33416 (8)	0.26892 (13)	0.0429 (3)	
C8	0.12327 (14)	0.23418 (9)	0.40514 (12)	0.0443 (3)	
C15	0.19303 (14)	0.25353 (8)	−0.03619 (12)	0.0422 (3)	
C10	0.02937 (14)	0.15529 (7)	−0.01043 (12)	0.0394 (3)	
C11	−0.06657 (16)	0.10293 (8)	−0.07638 (13)	0.0454 (3)	
C14	−0.07055 (15)	0.13790 (8)	0.17140 (13)	0.0433 (3)	
H14	−0.0734	0.1493	0.2540	0.052*	
O1	−0.05510 (14)	0.09050 (6)	−0.19682 (10)	0.0605 (3)	
C12	−0.16431 (17)	0.06843 (8)	−0.01514 (15)	0.0517 (4)	
H12	−0.2285	0.0328	−0.0563	0.062*	
C13	−0.16670 (17)	0.08690 (8)	0.10732 (15)	0.0516 (4)	
H13	−0.2346	0.0644	0.1471	0.062*	
C16	−0.13766 (19)	0.03015 (9)	−0.26109 (15)	0.0594 (4)	
H16A	−0.2410	0.0417	−0.2766	0.071*	
H16B	−0.1213	−0.0149	−0.2113	0.071*	
C7	0.00306 (18)	0.33991 (11)	0.48375 (16)	0.0592 (4)	
O5	−0.04221 (16)	0.37072 (9)	0.56792 (13)	0.0855 (5)	
C18	0.1006 (2)	0.24059 (13)	0.62469 (15)	0.0734 (5)	
H18A	0.1538	0.1948	0.6249	0.110*	
H18B	0.0079	0.2307	0.6464	0.110*	
H18C	0.1551	0.2743	0.6845	0.110*	
C17	−0.0879 (2)	0.01967 (12)	−0.38124 (18)	0.0775 (6)	
H17A	−0.1412	−0.0205	−0.4270	0.116*	
H17B	0.0144	0.0082	−0.3646	0.116*	
H17C	−0.1047	0.0646	−0.4297	0.116*	
C19	−0.1050 (2)	0.43883 (11)	0.3431 (2)	0.0813 (6)	
H19A	−0.1135	0.4538	0.2575	0.122*	
H19B	−0.0574	0.4774	0.3969	0.122*	
H19C	−0.2004	0.4302	0.3603	0.122*	
C1	0.0188 (2)	0.39517 (9)	−0.04694 (17)	0.0625 (4)	
H1A	−0.0728	0.4211	−0.0608	0.094*	
H1C	0.0328	0.3732	−0.1238	0.094*	
H1B	0.0964	0.4296	−0.0175	0.094*	

### Atomic displacement parameters (Å^2^)

**Table:**

	*U*^11^	*U*^22^	*U*^33^	*U*^12^	*U*^13^	*U*^23^
C4	0.0312 (5)	0.0409 (6)	0.0348 (6)	0.0038 (5)	0.0069 (5)	0.0008 (5)
O2	0.0530 (6)	0.0568 (6)	0.0407 (5)	−0.0071 (5)	0.0197 (4)	−0.0078 (4)
O4	0.0557 (6)	0.0419 (5)	0.0550 (6)	0.0095 (4)	0.0102 (5)	−0.0018 (4)
C3	0.0338 (6)	0.0433 (7)	0.0378 (6)	−0.0037 (5)	0.0056 (5)	0.0004 (5)
C5	0.0337 (6)	0.0455 (7)	0.0354 (6)	−0.0006 (5)	0.0063 (5)	−0.0050 (5)
N1	0.0478 (7)	0.0770 (9)	0.0351 (6)	−0.0090 (6)	0.0083 (5)	−0.0115 (6)
O3	0.0472 (6)	0.0811 (8)	0.0460 (6)	−0.0100 (5)	0.0172 (5)	0.0057 (5)
O6	0.0537 (6)	0.0729 (7)	0.0452 (6)	0.0102 (6)	0.0027 (5)	0.0103 (5)
C9	0.0362 (6)	0.0346 (6)	0.0373 (6)	0.0044 (5)	0.0082 (5)	−0.0003 (5)
N2	0.0510 (7)	0.0551 (8)	0.0589 (8)	0.0028 (6)	0.0140 (6)	−0.0192 (6)
C2	0.0408 (7)	0.0396 (7)	0.0446 (7)	0.0010 (5)	0.0035 (5)	0.0017 (5)
C6	0.0367 (6)	0.0453 (7)	0.0464 (7)	−0.0019 (5)	0.0073 (5)	−0.0100 (6)
C8	0.0331 (6)	0.0612 (9)	0.0368 (6)	−0.0047 (6)	0.0026 (5)	−0.0045 (6)
C15	0.0351 (6)	0.0540 (8)	0.0373 (6)	0.0001 (6)	0.0069 (5)	0.0043 (6)
C10	0.0416 (7)	0.0382 (6)	0.0402 (6)	0.0013 (5)	0.0126 (5)	−0.0017 (5)
C11	0.0527 (8)	0.0407 (7)	0.0418 (7)	0.0033 (6)	0.0071 (6)	−0.0053 (6)
C14	0.0458 (7)	0.0429 (7)	0.0433 (7)	−0.0003 (6)	0.0135 (6)	0.0008 (6)
O1	0.0773 (8)	0.0580 (7)	0.0462 (6)	−0.0103 (6)	0.0123 (5)	−0.0163 (5)
C12	0.0510 (8)	0.0416 (7)	0.0601 (9)	−0.0067 (6)	0.0052 (7)	−0.0045 (7)
C13	0.0511 (8)	0.0475 (8)	0.0588 (9)	−0.0084 (6)	0.0172 (7)	0.0019 (7)
C16	0.0656 (10)	0.0489 (8)	0.0585 (9)	0.0060 (7)	−0.0005 (7)	−0.0188 (7)
C7	0.0491 (8)	0.0773 (11)	0.0524 (9)	−0.0082 (8)	0.0126 (7)	−0.0248 (8)
O5	0.0845 (9)	0.1113 (11)	0.0661 (8)	0.0010 (8)	0.0283 (7)	−0.0401 (8)
C18	0.0725 (11)	0.1111 (16)	0.0368 (8)	−0.0087 (11)	0.0110 (8)	−0.0010 (9)
C17	0.0926 (14)	0.0744 (12)	0.0624 (11)	0.0085 (11)	0.0076 (10)	−0.0287 (10)
C19	0.0893 (14)	0.0655 (11)	0.0923 (15)	0.0240 (10)	0.0258 (11)	−0.0246 (11)
C1	0.0695 (11)	0.0531 (9)	0.0621 (10)	0.0046 (8)	0.0062 (8)	0.0156 (8)

### Geometric parameters (Å, º)

**Table:**

C4—C5	1.4926 (18)	C10—C11	1.3975 (19)
C4—C9	1.5157 (17)	C11—O1	1.3584 (17)
C4—C3	1.5263 (18)	C11—C12	1.381 (2)
C4—H4	0.9800	C14—C13	1.374 (2)
O2—C15	1.3552 (17)	C14—H14	0.9300
O2—C10	1.3972 (16)	O1—C16	1.4316 (18)
O4—C6	1.3373 (18)	C12—C13	1.382 (2)
O4—C2	1.4538 (17)	C12—H12	0.9300
C3—C15	1.5038 (19)	C13—H13	0.9300
C3—C2	1.5288 (18)	C16—C17	1.487 (3)
C3—H3	0.9800	C16—H16A	0.9700
C5—C6	1.3448 (19)	C16—H16B	0.9700
C5—C8	1.4301 (19)	C7—O5	1.2175 (19)
N1—C7	1.368 (2)	C18—H18A	0.9600
N1—C8	1.3926 (19)	C18—H18B	0.9600
N1—C18	1.464 (2)	C18—H18C	0.9600
O3—C15	1.1910 (16)	C17—H17A	0.9600
O6—C8	1.2197 (19)	C17—H17B	0.9600
C9—C10	1.3812 (18)	C17—H17C	0.9600
C9—C14	1.3832 (18)	C19—H19A	0.9600
N2—C6	1.3695 (18)	C19—H19B	0.9600
N2—C7	1.384 (2)	C19—H19C	0.9600
N2—C19	1.462 (2)	C1—H1A	0.9600
C2—C1	1.505 (2)	C1—H1C	0.9600
C2—H2	0.9800	C1—H1B	0.9600
			
C5—C4—C9	113.56 (10)	O1—C11—C10	116.37 (13)
C5—C4—C3	108.40 (11)	C12—C11—C10	118.13 (13)
C9—C4—C3	108.06 (10)	C13—C14—C9	120.06 (13)
C5—C4—H4	108.9	C13—C14—H14	120.0
C9—C4—H4	108.9	C9—C14—H14	120.0
C3—C4—H4	108.9	C11—O1—C16	117.45 (13)
C15—O2—C10	120.36 (10)	C11—C12—C13	119.93 (13)
C6—O4—C2	117.52 (10)	C11—C12—H12	120.0
C15—C3—C4	111.61 (11)	C13—C12—H12	120.0
C15—C3—C2	111.33 (11)	C14—C13—C12	121.25 (14)
C4—C3—C2	108.91 (10)	C14—C13—H13	119.4
C15—C3—H3	108.3	C12—C13—H13	119.4
C4—C3—H3	108.3	O1—C16—C17	107.20 (15)
C2—C3—H3	108.3	O1—C16—H16A	110.3
C6—C5—C8	119.19 (13)	C17—C16—H16A	110.3
C6—C5—C4	120.79 (12)	O1—C16—H16B	110.3
C8—C5—C4	120.02 (12)	C17—C16—H16B	110.3
C7—N1—C8	124.76 (13)	H16A—C16—H16B	108.5
C7—N1—C18	116.92 (14)	O5—C7—N1	122.64 (18)
C8—N1—C18	118.10 (16)	O5—C7—N2	121.11 (18)
C10—C9—C14	118.43 (12)	N1—C7—N2	116.25 (13)
C10—C9—C4	118.03 (11)	N1—C18—H18A	109.5
C14—C9—C4	123.51 (11)	N1—C18—H18B	109.5
C6—N2—C7	121.19 (14)	H18A—C18—H18B	109.5
C6—N2—C19	121.01 (15)	N1—C18—H18C	109.5
C7—N2—C19	117.55 (14)	H18A—C18—H18C	109.5
O4—C2—C1	106.06 (12)	H18B—C18—H18C	109.5
O4—C2—C3	108.82 (10)	C16—C17—H17A	109.5
C1—C2—C3	115.33 (13)	C16—C17—H17B	109.5
O4—C2—H2	108.8	H17A—C17—H17B	109.5
C1—C2—H2	108.8	C16—C17—H17C	109.5
C3—C2—H2	108.8	H17A—C17—H17C	109.5
O4—C6—C5	125.03 (13)	H17B—C17—H17C	109.5
O4—C6—N2	112.57 (12)	N2—C19—H19A	109.5
C5—C6—N2	122.39 (14)	N2—C19—H19B	109.5
O6—C8—N1	120.23 (13)	H19A—C19—H19B	109.5
O6—C8—C5	123.69 (13)	N2—C19—H19C	109.5
N1—C8—C5	116.08 (13)	H19A—C19—H19C	109.5
O3—C15—O2	117.79 (13)	H19B—C19—H19C	109.5
O3—C15—C3	124.45 (13)	C2—C1—H1A	109.5
O2—C15—C3	117.76 (11)	C2—C1—H1C	109.5
C9—C10—O2	122.01 (12)	H1A—C1—H1C	109.5
C9—C10—C11	122.15 (13)	C2—C1—H1B	109.5
O2—C10—C11	115.83 (12)	H1A—C1—H1B	109.5
O1—C11—C12	125.50 (13)	H1C—C1—H1B	109.5
			
C5—C4—C3—C15	−175.85 (10)	C4—C5—C8—N1	179.44 (11)
C9—C4—C3—C15	−52.37 (13)	C10—O2—C15—O3	178.55 (12)
C5—C4—C3—C2	−52.53 (13)	C10—O2—C15—C3	−1.65 (18)
C9—C4—C3—C2	70.95 (13)	C4—C3—C15—O3	−141.90 (14)
C9—C4—C5—C6	−96.05 (15)	C2—C3—C15—O3	96.17 (16)
C3—C4—C5—C6	24.06 (16)	C4—C3—C15—O2	38.32 (15)
C9—C4—C5—C8	84.60 (14)	C2—C3—C15—O2	−83.61 (14)
C3—C4—C5—C8	−155.30 (11)	C14—C9—C10—O2	178.53 (12)
C5—C4—C9—C10	155.04 (11)	C4—C9—C10—O2	0.45 (18)
C3—C4—C9—C10	34.75 (15)	C14—C9—C10—C11	−2.3 (2)
C5—C4—C9—C14	−22.92 (17)	C4—C9—C10—C11	179.67 (12)
C3—C4—C9—C14	−143.22 (12)	C15—O2—C10—C9	−19.32 (19)
C6—O4—C2—C1	−165.09 (12)	C15—O2—C10—C11	161.42 (12)
C6—O4—C2—C3	−40.44 (15)	C9—C10—C11—O1	−179.05 (12)
C15—C3—C2—O4	−174.74 (11)	O2—C10—C11—O1	0.21 (18)
C4—C3—C2—O4	61.77 (13)	C9—C10—C11—C12	1.0 (2)
C15—C3—C2—C1	−55.75 (16)	O2—C10—C11—C12	−179.70 (13)
C4—C3—C2—C1	−179.23 (12)	C10—C9—C14—C13	1.5 (2)
C2—O4—C6—C5	10.88 (19)	C4—C9—C14—C13	179.43 (13)
C2—O4—C6—N2	−170.26 (11)	C12—C11—O1—C16	−8.5 (2)
C8—C5—C6—O4	177.18 (12)	C10—C11—O1—C16	171.64 (13)
C4—C5—C6—O4	−2.2 (2)	O1—C11—C12—C13	−178.93 (14)
C8—C5—C6—N2	−1.6 (2)	C10—C11—C12—C13	1.0 (2)
C4—C5—C6—N2	179.07 (12)	C9—C14—C13—C12	0.5 (2)
C7—N2—C6—O4	−178.82 (13)	C11—C12—C13—C14	−1.7 (2)
C19—N2—C6—O4	7.1 (2)	C11—O1—C16—C17	−170.40 (14)
C7—N2—C6—C5	0.1 (2)	C8—N1—C7—O5	175.43 (15)
C19—N2—C6—C5	−174.03 (15)	C18—N1—C7—O5	0.9 (2)
C7—N1—C8—O6	−177.10 (14)	C8—N1—C7—N2	−4.5 (2)
C18—N1—C8—O6	−2.6 (2)	C18—N1—C7—N2	−179.10 (14)
C7—N1—C8—C5	3.1 (2)	C6—N2—C7—O5	−177.11 (15)
C18—N1—C8—C5	177.62 (13)	C19—N2—C7—O5	−2.8 (2)
C6—C5—C8—O6	−179.70 (13)	C6—N2—C7—N1	2.9 (2)
C4—C5—C8—O6	−0.3 (2)	C19—N2—C7—N1	177.15 (15)
C6—C5—C8—N1	0.07 (19)		

### Hydrogen-bond geometry (Å, º)

**Table:**

*D*—H···*A*	*D*—H	H···*A*	*D*···*A*	*D*—H···*A*
C2—H2···O6^i^	0.98	2.40	3.1480 (17)	132
C18—H18*C*···O3^ii^	0.96	2.45	3.252 (2)	140

Symmetry codes: (i) *x*−1/2, −*y*+1/2, *z*−1/2; (ii) *x*, *y*, *z*+1.
